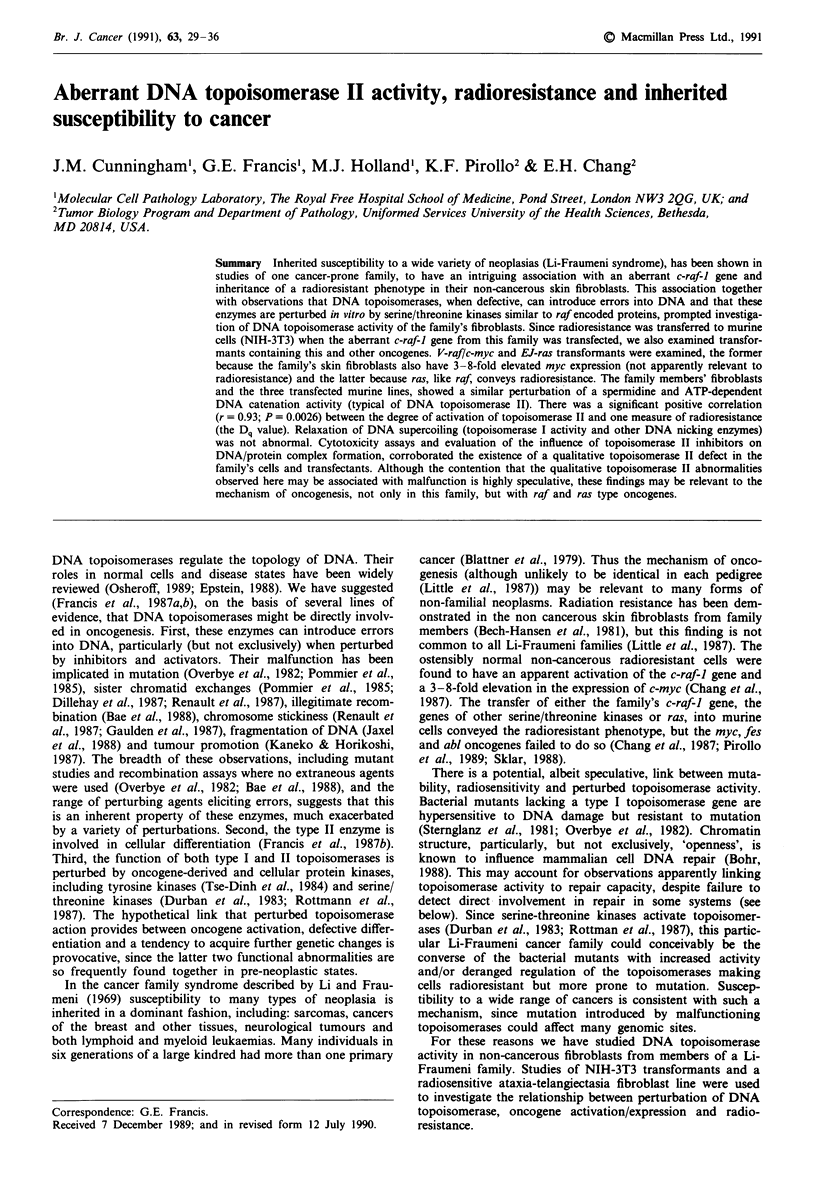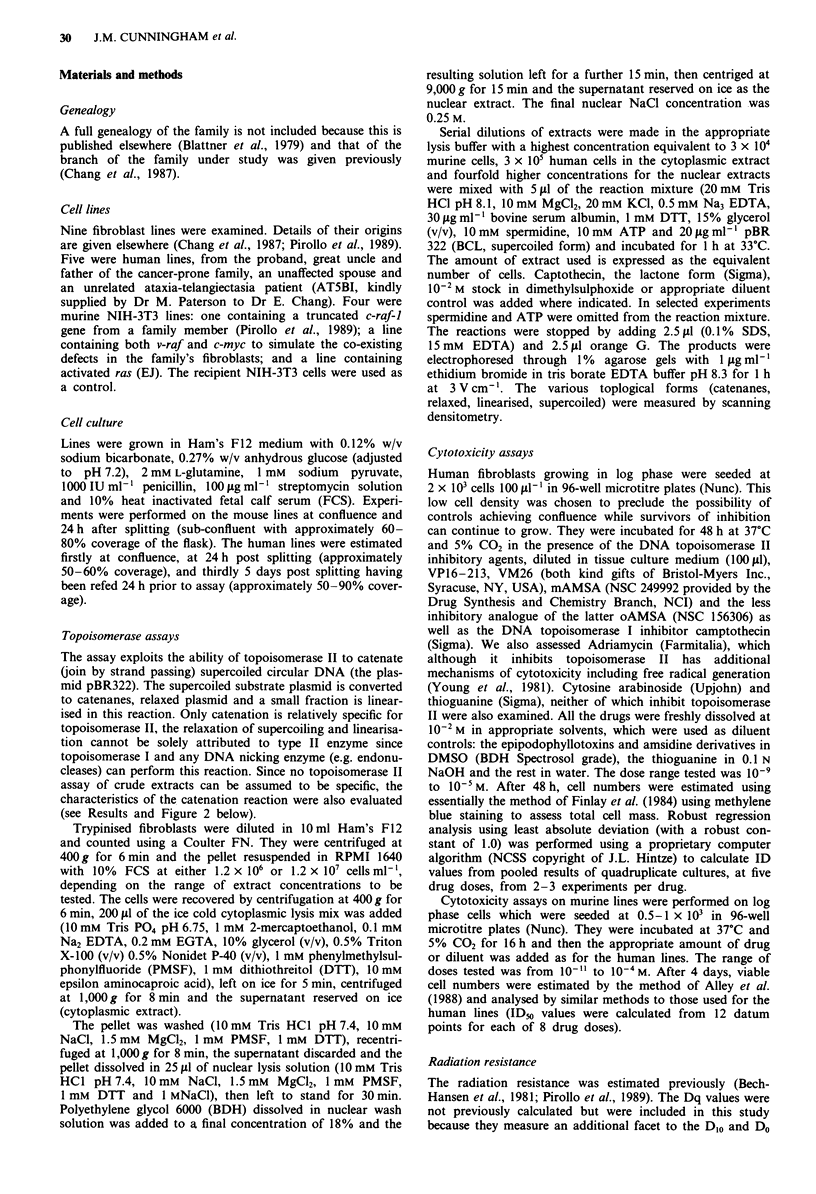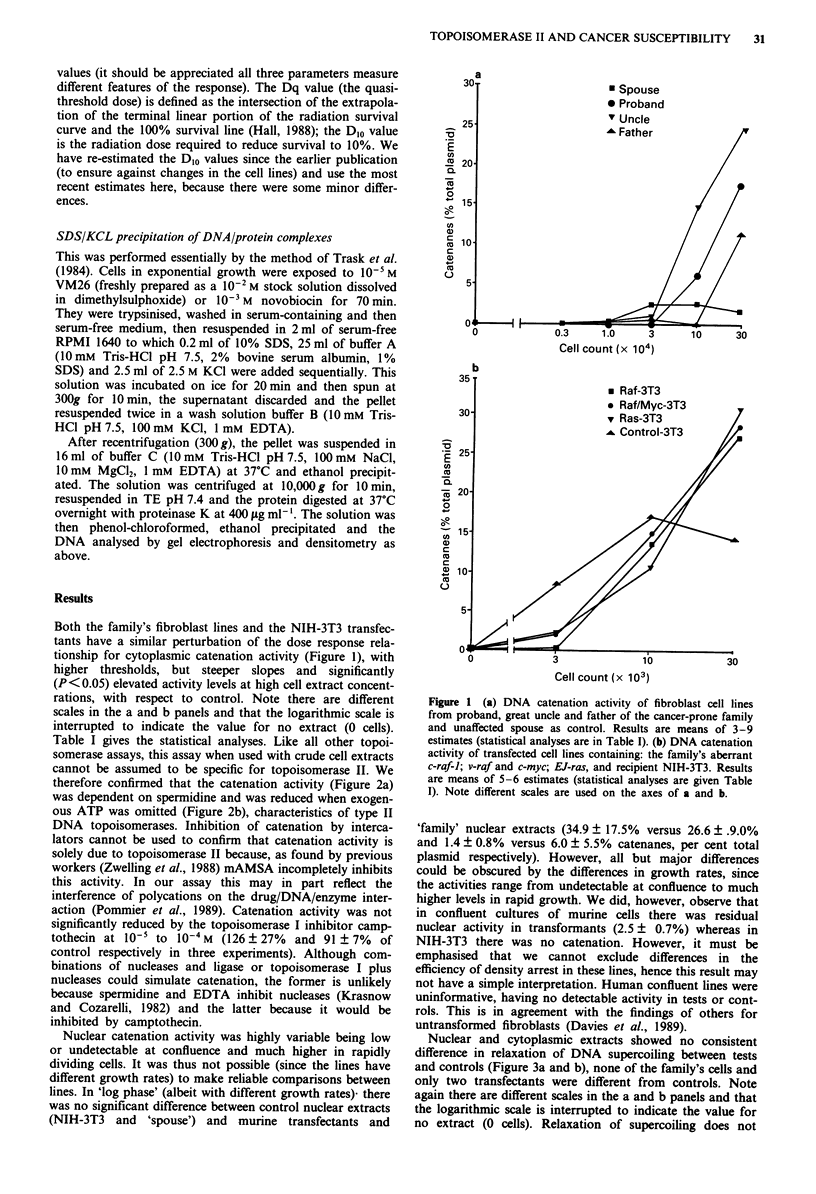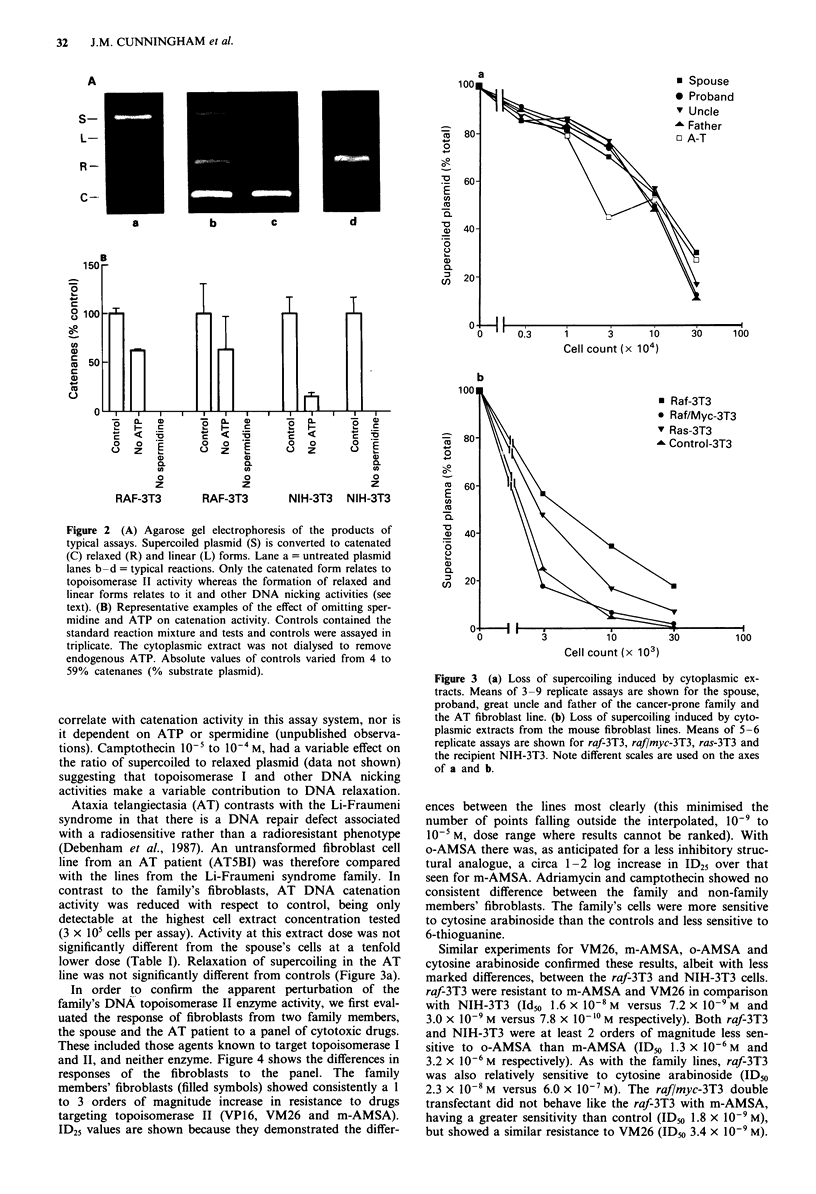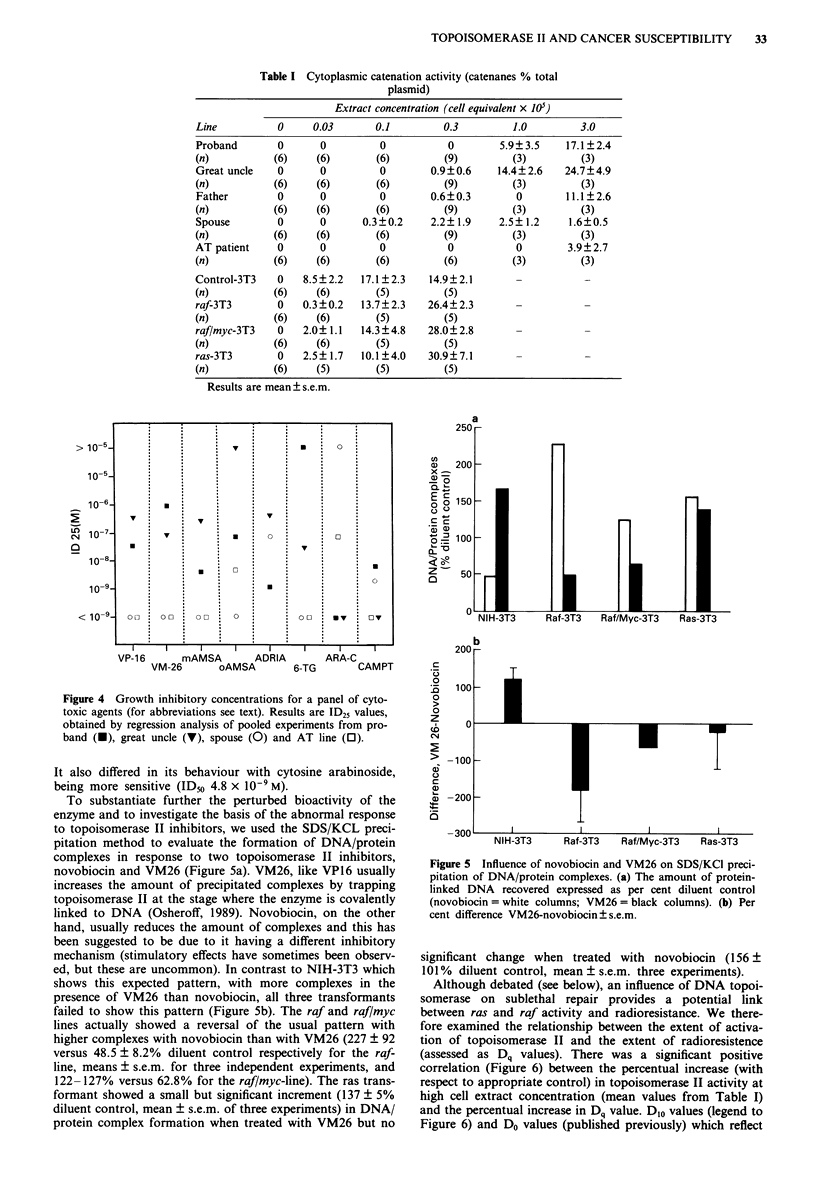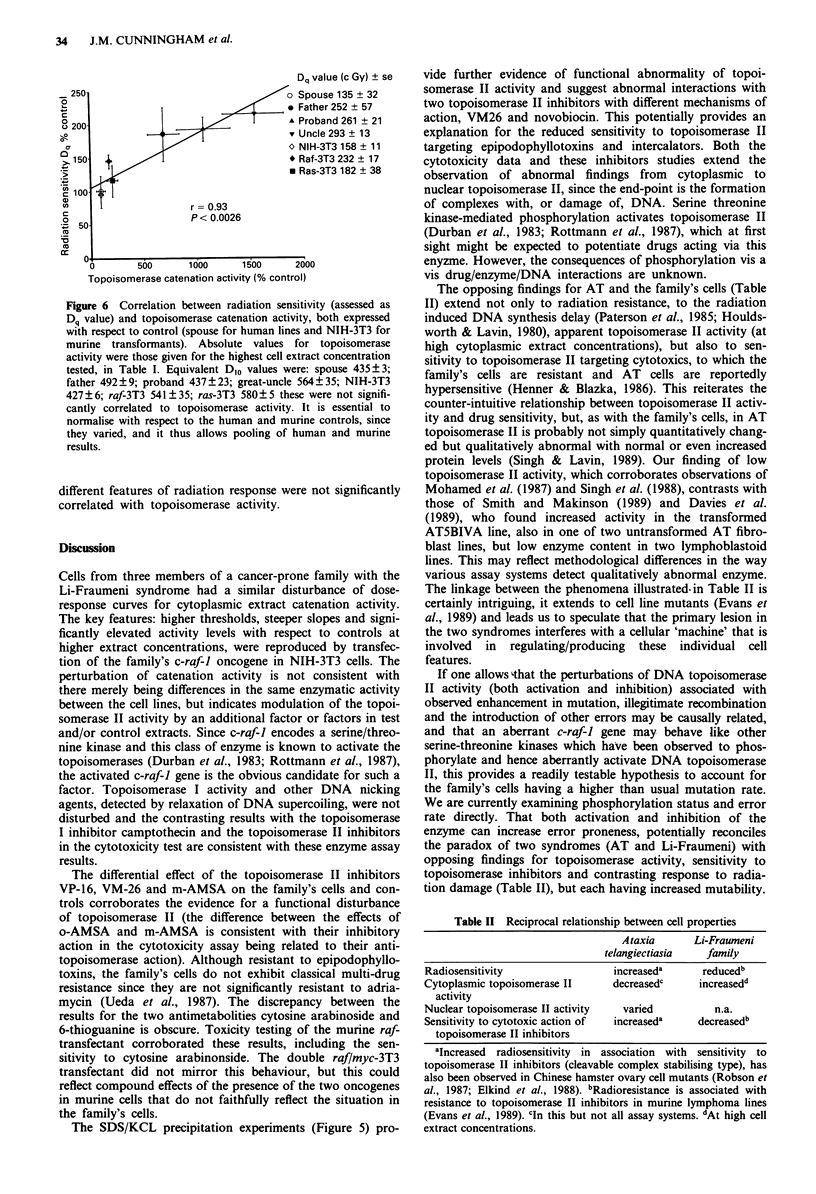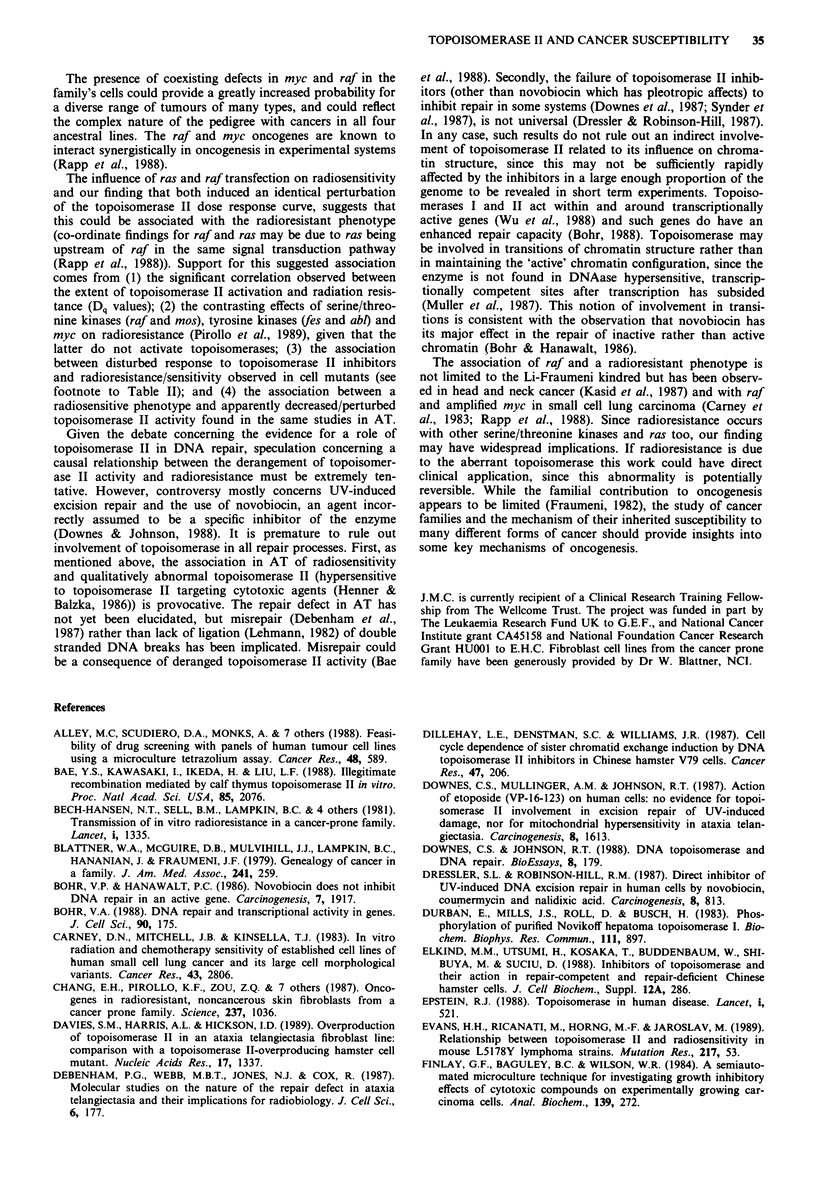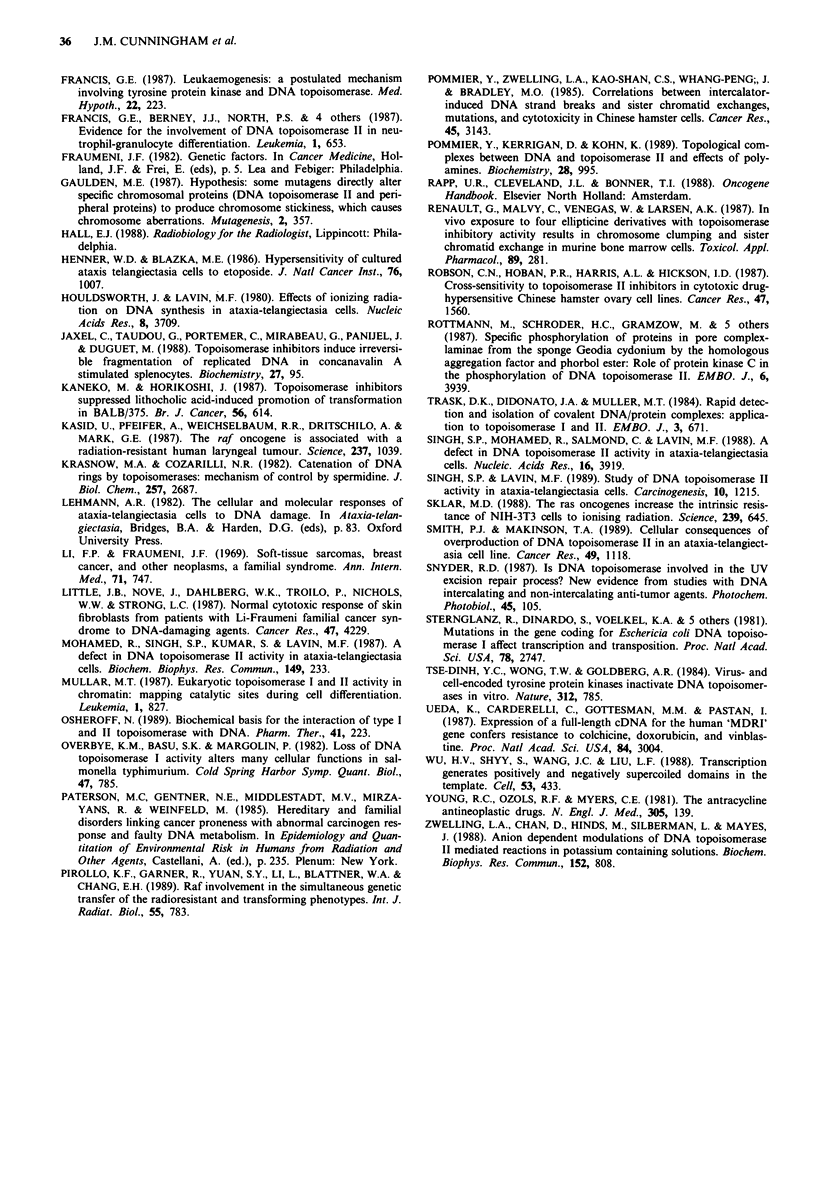# Aberrant DNA topoisomerase II activity, radioresistance and inherited susceptibility to cancer.

**DOI:** 10.1038/bjc.1991.8

**Published:** 1991-01

**Authors:** J. M. Cunningham, G. E. Francis, M. J. Holland, K. F. Pirollo, E. H. Chang

**Affiliations:** Molecular Cell Pathology Laboratory, Royal Free Hospital School of Medicine, London, UK.

## Abstract

**Images:**


Br. J. Cancer (1991), 63, 29-36 © Macmillan Press Ltd., 1991

### Aberrant DNA topoisomerase II activity, radioresistance and inherited susceptibility to cancer

J.M. Cunningham[1], G.E. Francis[1], M.J. Holland[1], K.F. Pirollo[2] & E.H. Chang[2]

[1]*Molecular Cell Pathology Laboratory, The Royal Free Hospital School of Medicine, Pond Street, London NW3 2QG, UK; and* [2]*Tumor Biology Program and Department of Pathology, Uniformed Services University of the Health Sciences, Bethesda, MD 20814, USA.*

**Summary** Inherited susceptibility to a wide variety of neoplasias (Li-Fraumeni syndrome), has been shown in studies of one cancer-prone family, to have an intriguing association with an aberrant *c-raf-1* gene and inheritance of a radioresistant phenotype in their non-cancerous skin fibroblasts. This association together with observations that DNA topoisomerases, when defective, can introduce errors into DNA and that these enzymes are perturbed *in vitro* by serine/threonine kinases similar to *raf* encoded proteins, prompted investigation of DNA topoisomerase activity of the family's fibroblasts. Since radioresistance was transferred to murine cells (NIH-3T3) when the aberrant *c-raf-1* gene from this family was transfected, we also examined transformants containing this and other oncogenes. *V-raf/c-myc* and *EJ-ras* transformants were examined, the former because the family's skin fibroblasts also have 3-8-fold elevated *myc* expression (not apparently relevant to radioresistance) and the latter because *ras*, like *raf*, conveys radioresistance. The family members' fibroblasts and the three transfected murine lines, showed a similar perturbation of a spermidine and ATP-dependent DNA catenation activity (typical of DNA topoisomerase II). There was a significant positive correlation ($r = 0.93$; $P = 0.0026$) between the degree of activation of topoisomerase II and one measure of radioresistance (the $D_q$ value). Relaxation of DNA supercoiling (topoisomerase I activity and other DNA nicking enzymes) was not abnormal. Cytotoxicity assays and evaluation of the influence of topoisomerase II inhibitors on DNA/protein complex formation, corroborated the existence of a qualitative topoisomerase II defect in the family's cells and transfectants. Although the contention that the qualitative topoisomerase II abnormalities observed here may be associated with malfunction is highly speculative, these findings may be relevant to the mechanism of oncogenesis, not only in this family, but with *raf* and *ras* type oncogenes.

DNA topoisomerases regulate the topology of DNA. Their roles in normal cells and disease states have been widely reviewed (Osheroff, 1989; Epstein, 1988). We have suggested (Francis *et al.*, 1987*a,b*), on the basis of several lines of evidence, that DNA topoisomerases might be directly involved in oncogenesis. First, these enzymes can introduce errors into DNA, particularly (but not exclusively) when perturbed by inhibitors and activators. Their malfunction has been implicated in mutation (Overbye *et al.*, 1982; Pommier *et al.*, 1985), sister chromatid exchanges (Pommier *et al.*, 1985; Dillehay *et al.*, 1987; Renault *et al.*, 1987), illegitimate recombination (Bae *et al.*, 1988), chromosome stickiness (Renault *et al.*, 1987; Gaulden *et al.*, 1987), fragmentation of DNA (Jaxel *et al.*, 1988) and tumour promotion (Kaneko & Horikoshi, 1987). The breadth of these observations, including mutant studies and recombination assays where no extraneous agents were used (Overbye *et al.*, 1982; Bae *et al.*, 1988), and the range of perturbing agents eliciting errors, suggests that this is an inherent property of these enzymes, much exacerbated by a variety of perturbations. Second, the type II enzyme is involved in cellular differentiation (Francis *et al.*, 1987*b*). Third, the function of both type I and II topoisomerases is perturbed by oncogene-derived and cellular protein kinases, including tyrosine kinases (Tse-Dinh *et al.*, 1984) and serine/threonine kinases (Durban *et al.*, 1983; Rottmann *et al.*, 1987). The hypothetical link that perturbed topoisomerase action provides between oncogene activation, defective differentiation and a tendency to acquire further genetic changes is provocative, since the latter two functional abnormalities are so frequently found together in pre-neoplastic states.

In the cancer family syndrome described by Li and Fraumeni (1969) susceptibility to many types of neoplasia is inherited in a dominant fashion, including: sarcomas, cancers of the breast and other tissues, neurological tumours and both lymphoid and myeloid leukaemias. Many individuals in six generations of a large kindred had more than one primary cancer (Blattner *et al.*, 1979). Thus the mechanism of oncogenesis (although unlikely to be identical in each pedigree (Little *et al.*, 1987)) may be relevant to many forms of non-familial neoplasms. Radiation resistance has been demonstrated in the non cancerous skin fibroblasts from family members (Bech-Hansen *et al.*, 1981), but this finding is not common to all Li-Fraumeni families (Little *et al.*, 1987). The ostensibly normal non-cancerous radioresistant cells were found to have an apparent activation of the *c-raf-1* gene and a 3-8-fold elevation in the expression of *c-myc* (Chang *et al.*, 1987). The transfer of either the family's *c-raf-1* gene, the genes of other serine/threonine kinases or *ras*, into murine cells conveyed the radioresistant phenotype, but the *myc*, *fes* and *abl* oncogenes failed to do so (Chang *et al.*, 1987; Pirollo *et al.*, 1989; Sklar, 1988).

There is a potential, albeit speculative, link between mutability, radiosensitivity and perturbed topoisomerase activity. Bacterial mutants lacking a type I topoisomerase gene are hypersensitive to DNA damage but resistant to mutation (Sternglanz *et al.*, 1981; Overbye *et al.*, 1982). Chromatin structure, particularly, but not exclusively, 'openness', is known to influence mammalian cell DNA repair (Bohr, 1988). This may account for observations apparently linking topoisomerase activity to repair capacity, despite failure to detect direct involvement in repair in some systems (see below). Since serine-threonine kinases activate topoisomerases (Durban *et al.*, 1983; Rottman *et al.*, 1987), this particular Li-Fraumeni cancer family could conceivably be the converse of the bacterial mutants with increased activity and/or deranged regulation of the topoisomerases making cells radioresistant but more prone to mutation. Susceptibility to a wide range of cancers is consistent with such a mechanism, since mutation introduced by malfunctioning topoisomerases could affect many genomic sites.

For these reasons we have studied DNA topoisomerase activity in non-cancerous fibroblasts from members of a Li-Fraumeni family. Studies of NIH-3T3 transformants and a radiosensitive ataxia-telangiectasia fibroblast line were used to investigate the relationship between perturbation of DNA topoisomerase, oncogene activation/expression and radioresistance.

Correspondence: G.E. Francis.
Received 7 December 1989; and in revised form 12 July 1990.

### Materials and methods

### *Genealogy*

A full genealogy of the family is not included because this is published elsewhere (Blattner *et al.*, 1979) and that of the branch of the family under study was given previously (Chang *et al.*, 1987).

### *Cell lines*

Nine fibroblast lines were examined. Details of their origins are given elsewhere (Chang *et al.*, 1987; Pirollo *et al.*, 1989). Five were human lines, from the proband, great uncle and father of the cancer-prone family, an unaffected spouse and an unrelated ataxia-telangiectasia patient (AT5BI, kindly supplied by Dr M. Paterson to Dr E. Chang). Four were murine NIH-3T3 lines: one containing a truncated *c-raf-1* gene from a family member (Pirollo *et al.*, 1989); a line containing both *v-raf* and *c-myc* to simulate the co-existing defects in the family's fibroblasts; and a line containing activated *ras* (EJ). The recipient NIH-3T3 cells were used as a control.

### *Cell culture*

Lines were grown in Ham's F12 medium with 0.12% w/v sodium bicarbonate, 0.27% w/v anhydrous glucose (adjusted to pH 7.2), 2 mM L-glutamine, 1 mM sodium pyruvate, 1000 IU $ml^{-1}$ penicillin, 100 $\mu g\ ml^{-1}$ streptomycin solution and 10% heat inactivated fetal calf serum (FCS). Experiments were performed on the mouse lines at confluence and 24 h after splitting (sub-confluent with approximately 60–80% coverage of the flask). The human lines were estimated firstly at confluence, at 24 h post splitting (approximately 50–60% coverage), and thirdly 5 days post splitting having been refed 24 h prior to assay (approximately 50–90% coverage).

### *Topoisomerase assays*

The assay exploits the ability of topoisomerase II to catenate (join by strand passing) supercoiled circular DNA (the plasmid pBR322). The supercoiled substrate plasmid is converted to catenanes, relaxed plasmid and a small fraction is linearised in this reaction. Only catenation is relatively specific for topoisomerase II, the relaxation of supercoiling and linearisation cannot be solely attributed to type II enzyme since topoisomerase I and any DNA nicking enzyme (e.g. endonucleases) can perform this reaction. Since no topoisomerase II assay of crude extracts can be assumed to be specific, the characteristics of the catenation reaction were also evaluated (see Results and Figure 2 below).

Trypinised fibroblasts were diluted in 10 ml Ham's F12 and counted using a Coulter FN. They were centrifuged at 400 *g* for 6 min and the pellet resuspended in RPMI 1640 with 10% FCS at either $1.2 \times 10^6$ or $1.2 \times 10^7$ cells $ml^{-1}$, depending on the range of extract concentrations to be tested. The cells were recovered by centrifugation at 400 *g* for 6 min, 200 µl of the ice cold cytoplasmic lysis mix was added (10 mM Tris $PO_4$ pH 6.75, 1 mM 2-mercaptoethanol, 0.1 mM $Na_2$ EDTA, 0.2 mM EGTA, 10% glycerol (v/v), 0.5% Triton X-100 (v/v) 0.5% Nonidet P-40 (v/v), 1 mM phenylmethylsulphonylfluoride (PMSF), 1 mM dithiothreitol (DTT), 10 mM epsilon aminocaproic acid), left on ice for 5 min, centrifuged at 1,000 *g* for 8 min and the supernatant reserved on ice (cytoplasmic extract).

The pellet was washed (10 mM Tris HC1 pH 7.4, 10 mM NaCl, 1.5 mM $MgCl_2$, 1 mM PMSF, 1 mM DTT), recentrifuged at 1,000 *g* for 8 min, the supernatant discarded and the pellet dissolved in 25 µl of nuclear lysis solution (10 mM Tris HCl pH 7.4, 10 mM NaCl, 1.5 mM $MgCl_2$, 1 mM PMSF, 1 mM DTT and 1 MNaCl), then left to stand for 30 min. Polyethylene glycol 6000 (BDH) dissolved in nuclear wash solution was added to a final concentration of 18% and the resulting solution left for a further 15 min, then centriged at 9,000 *g* for 15 min and the supernatant reserved on ice as the nuclear extract. The final nuclear NaCl concentration was 0.25 M.

Serial dilutions of extracts were made in the appropriate lysis buffer with a highest concentration equivalent to $3 \times 10^4$ murine cells, $3 \times 10^5$ human cells in the cytoplasmic extract and fourfold higher concentrations for the nuclear extracts were mixed with 5 µl of the reaction mixture (20 mM Tris HCl pH 8.1, 10 mM $MgCl_2$, 20 mM KCl, 0.5 mM $Na_3$ EDTA, 30 $\mu g\ ml^{-1}$ bovine serum albumin, 1 mM DTT, 15% glycerol (v/v), 10 mM spermidine, 10 mM ATP and 20 $\mu g\ ml^{-1}$ pBR 322 (BCL, supercoiled form) and incubated for 1 h at 33°C. The amount of extract used is expressed as the equivalent number of cells. Captothecin, the lactone form (Sigma), $10^{-2}$ M stock in dimethylsulphoxide or appropriate diluent control was added where indicated. In selected experiments spermidine and ATP were omitted from the reaction mixture. The reactions were stopped by adding 2.5 µl (0.1% SDS, 15 mM EDTA) and 2.5 µl orange G. The products were electrophoresed through 1% agarose gels with 1 $\mu g\ ml^{-1}$ ethidium bromide in tris borate EDTA buffer pH 8.3 for 1 h at 3 V $cm^{-1}$. The various toplogical forms (catenanes, relaxed, linearised, supercoiled) were measured by scanning densitometry.

### *Cytotoxicity assays*

Human fibroblasts growing in log phase were seeded at $2 \times 10^3$ cells 100 $\mu l^{-1}$ in 96-well microtitre plates (Nunc). This low cell density was chosen to preclude the possibility of controls achieving confluence while survivors of inhibition can continue to grow. They were incubated for 48 h at 37°C and 5% $CO_2$ in the presence of the DNA topoisomerase II inhibitory agents, diluted in tissue culture medium (100 µl), VP16-213, VM26 (both kind gifts of Bristol-Myers Inc., Syracuse, NY, USA), mAMSA (NSC 249992 provided by the Drug Synthesis and Chemistry Branch, NCI) and the less inhibitory analogue of the latter oAMSA (NSC 156306) as well as the DNA topoisomerase I inhibitor camptothecin (Sigma). We also assessed Adriamycin (Farmitalia), which although it inhibits topoisomerase II has additional mechanisms of cytotoxicity including free radical generation (Young *et al.*, 1981). Cytosine arabinoside (Upjohn) and thioguanine (Sigma), neither of which inhibit topoisomerase II were also examined. All the drugs were freshly dissolved at $10^{-2}$ M in appropriate solvents, which were used as diluent controls: the epipodophyllotoxins and amsidine derivatives in DMSO (BDH Spectrosol grade), the thioguanine in 0.1 N NaOH and the rest in water. The dose range tested was $10^{-9}$ to $10^{-5}$ M. After 48 h, cell numbers were estimated using essentially the method of Finlay *et al.* (1984) using methylene blue staining to assess total cell mass. Robust regression analysis using least absolute deviation (with a robust constant of 1.0) was performed using a proprietary computer algorithm (NCSS copyright of J.L. Hintze) to calculate ID values from pooled results of quadruplicate cultures, at five drug doses, from 2–3 experiments per drug.

Cytotoxicity assays on murine lines were performed on log phase cells which were seeded at $0.5-1 \times 10^3$ in 96-well microtitre plates (Nunc). They were incubated at 37°C and 5% $CO_2$ for 16 h and then the appropriate amount of drug or diluent was added as for the human lines. The range of doses tested was from $10^{-11}$ to $10^{-4}$ M. After 4 days, viable cell numbers were estimated by the method of Alley *et al.* (1988) and analysed by similar methods to those used for the human lines ($ID_{50}$ values were calculated from 12 datum points for each of 8 drug doses).

### *Radiation resistance*

The radiation resistance was estimated previously (Bech-Hansen *et al.*, 1981; Pirollo *et al.*, 1989). The Dq values were not previously calculated but were included in this study because they measure an additional facet to the $D_{10}$ and $D_0$

values (it should be appreciated all three parameters measure different features of the response). The Dq value (the quasi-threshold dose) is defined as the intersection of the extrapolation of the terminal linear portion of the radiation survival curve and the 100% survival line (Hall, 1988); the $D_{10}$ value is the radiation dose required to reduce survival to 10%. We have re-estimated the $D_{10}$ values since the earlier publication (to ensure against changes in the cell lines) and use the most recent estimates here, because there were some minor differences.

*SDS/KCL precipitation of DNA/protein complexes*

This was performed essentially by the method of Trask *et al.* (1984). Cells in exponential growth were exposed to $10^{-5}$ M VM26 (freshly prepared as a $10^{-2}$ M stock solution dissolved in dimethylsulphoxide) or $10^{-3}$ M novobiocin for 70 min. They were trypsinised, washed in serum-containing and then serum-free medium, then resuspended in 2 ml of serum-free RPMI 1640 to which 0.2 ml of 10% SDS, 25 ml of buffer A (10 mM Tris-HCl pH 7.5, 2% bovine serum albumin, 1% SDS) and 2.5 ml of 2.5 M KCl were added sequentially. This solution was incubated on ice for 20 min and then spun at 300*g* for 10 min, the supernatant discarded and the pellet resuspended twice in a wash solution buffer B (10 mM Tris-HCl pH 7.5, 100 mM KCl, 1 mM EDTA).

After recentrifugation (300 *g*), the pellet was suspended in 16 ml of buffer C (10 mM Tris-HCl pH 7.5, 100 mM NaCl, 10 mM $MgCl_2$, 1 mM EDTA) at 37°C and ethanol precipitated. The solution was centrifuged at 10,000 *g* for 10 min, resuspended in TE pH 7.4 and the protein digested at 37°C overnight with proteinase K at 400 $\mu$g ml$^{-1}$. The solution was then phenol-chloroformed, ethanol precipitated and the DNA analysed by gel electrophoresis and densitometry as above.

### Results

Both the family's fibroblast lines and the NIH-3T3 transfectants have a similar perturbation of the dose response relationship for cytoplasmic catenation activity (Figure 1), with higher thresholds, but steeper slopes and significantly ($P<0.05$) elevated activity levels at high cell extract concentrations, with respect to control. Note there are different scales in the a and b panels and that the logarithmic scale is interrupted to indicate the value for no extract (0 cells). Table I gives the statistical analyses. Like all other topoisomerase assays, this assay when used with crude cell extracts cannot be assumed to be specific for topoisomerase II. We therefore confirmed that the catenation activity (Figure 2a) was dependent on spermidine and was reduced when exogenous ATP was omitted (Figure 2b), characteristics of type II DNA topoisomerases. Inhibition of catenation by intercalators cannot be used to confirm that catenation activity is solely due to topoisomerase II because, as found by previous workers (Zwelling *et al.*, 1988) mAMSA incompletely inhibits this activity. In our assay this may in part reflect the interference of polycations on the drug/DNA/enzyme interaction (Pommier *et al.*, 1989). Catenation activity was not significantly reduced by the topoisomerase I inhibitor camptothecin at $10^{-5}$ to $10^{-4}$ M ($126 \pm 27\%$ and $91 \pm 7\%$ of control respectively in three experiments). Although combinations of nucleases and ligase or topoisomerase I plus nucleases could simulate catenation, the former is unlikely because spermidine and EDTA inhibit nucleases (Krasnow and Cozarelli, 1982) and the latter because it would be inhibited by camptothecin.

Nuclear catenation activity was highly variable being low or undetectable at confluence and much higher in rapidly dividing cells. It was thus not possible (since the lines have different growth rates) to make reliable comparisons between lines. In 'log phase' (albeit with different growth rates)· there was no significant difference between control nuclear extracts (NIH-3T3 and 'spouse') and murine transfectants and

**Figure 1** (**a**) DNA catenation activity of fibroblast cell lines from proband, great uncle and father of the cancer-prone family and unaffected spouse as control. Results are means of 3–9 estimates (statistical analyses are in Table I). (**b**) DNA catenation activity of transfected cell lines containing: the family's aberrant *c-raf-1*; *v-raf* and *c-myc*; *EJ-ras*, and recipient NIH-3T3. Results are means of 5–6 estimates (statistical analyses are given Table I). Note different scales are used on the axes of **a** and **b**.

'family' nuclear extracts ($34.9 \pm 17.5\%$ versus $26.6 \pm .9.0\%$ and $1.4 \pm 0.8\%$ versus $6.0 \pm 5.5\%$ catenanes, per cent total plasmid respectively). However, all but major differences could be obscured by the differences in growth rates, since the activities range from undetectable at confluence to much higher levels in rapid growth. We did, however, observe that in confluent cultures of murine cells there was residual nuclear activity in transformants ($2.5 \pm$ 0.7%) whereas in NIH-3T3 there was no catenation. However, it must be emphasised that we cannot exclude differences in the efficiency of density arrest in these lines, hence this result may not have a simple interpretation. Human confluent lines were uninformative, having no detectable activity in tests or controls. This is in agreement with the findings of others for untransformed fibroblasts (Davies *et al.*, 1989).

Nuclear and cytoplasmic extracts showed no consistent difference in relaxation of DNA supercoiling between tests and controls (Figure 3a and b), none of the family's cells and only two transfectants were different from controls. Note again there are different scales in the a and b panels and that the logarithmic scale is interrupted to indicate the value for no extract (0 cells). Relaxation of supercoiling does not

**Figure 2** (**A**) Agarose gel electrophoresis of the products of typical assays. Supercoiled plasmid (S) is converted to catenated (C) relaxed (R) and linear (L) forms. Lane a = untreated plasmid lanes b–d = typical reactions. Only the catenated form relates to topoisomerase II activity whereas the formation of relaxed and linear forms relates to it and other DNA nicking activities (see text). (**B**) Representative examples of the effect of omitting spermidine and ATP on catenation activity. Controls contained the standard reaction mixture and tests and controls were assayed in triplicate. The cytoplasmic extract was not dialysed to remove endogenous ATP. Absolute values of controls varied from 4 to 59% catenanes (% substrate plasmid).

correlate with catenation activity in this assay system, nor is it dependent on ATP or spermidine (unpublished observations). Camptothecin $10^{-5}$ to $10^{-4}$ M, had a variable effect on the ratio of supercoiled to relaxed plasmid (data not shown) suggesting that topoisomerase I and other DNA nicking activities make a variable contribution to DNA relaxation.

Ataxia telangiectasia (AT) contrasts with the Li-Fraumeni syndrome in that there is a DNA repair defect associated with a radiosensitive rather than a radioresistant phenotype (Debenham *et al.*, 1987). An untransformed fibroblast cell line from an AT patient (AT5BI) was therefore compared with the lines from the Li-Fraumeni syndrome family. In contrast to the family's fibroblasts, AT DNA catenation activity was reduced with respect to control, being only detectable at the highest cell extract concentration tested ($3 \times 10^5$ cells per assay). Activity at this extract dose was not significantly different from the spouse's cells at a tenfold lower dose (Table I). Relaxation of supercoiling in the AT line was not significantly different from controls (Figure 3a).

In order to confirm the apparent perturbation of the family's DNA topoisomerase II enzyme activity, we first evaluated the response of fibroblasts from two family members, the spouse and the AT patient to a panel of cytotoxic drugs. These included those agents known to target topoisomerase I and II, and neither enzyme. Figure 4 shows the differences in responses of the fibroblasts to the panel. The family members' fibroblasts (filled symbols) showed consistently a 1 to 3 orders of magnitude increase in resistance to drugs targeting topoisomerase II (VP16, VM26 and m-AMSA). $ID_{25}$ values are shown because they demonstrated the differ-

**Figure 3** (**a**) Loss of supercoiling induced by cytoplasmic extracts. Means of 3–9 replicate assays are shown for the spouse, proband, great uncle and father of the cancer-prone family and the AT fibroblast line. (**b**) Loss of supercoiling induced by cytoplasmic extracts from the mouse fibroblast lines. Means of 5–6 replicate assays are shown for *raf*-3T3, *raf/myc*-3T3, *ras*-3T3 and the recipient NIH-3T3. Note different scales are used on the axes of **a** and **b**.

ences between the lines most clearly (this minimised the number of points falling outside the interpolated, $10^{-9}$ to $10^{-5}$ M, dose range where results cannot be ranked). With o-AMSA there was, as anticipated for a less inhibitory structural analogue, a circa 1–2 log increase in $ID_{25}$ over that seen for m-AMSA. Adriamycin and camptothecin showed no consistent difference between the family and non-family members' fibroblasts. The family's cells were more sensitive to cytosine arabinoside than the controls and less sensitive to 6-thioguanine.

Similar experiments for VM26, m-AMSA, o-AMSA and cytosine arabinoside confirmed these results, albeit with less marked differences, between the *raf*-3T3 and NIH-3T3 cells. *raf*-3T3 were resistant to m-AMSA and VM26 in comparison with NIH-3T3 ($Id_{50}$ $1.6 \times 10^{-8}$ M versus $7.2 \times 10^{-9}$ M and $3.0 \times 10^{-9}$ M versus $7.8 \times 10^{-10}$ M respectively). Both *raf*-3T3 and NIH-3T3 were at least 2 orders of magnitude less sensitive to o-AMSA than m-AMSA ($ID_{50}$ $1.3 \times 10^{-6}$ M and $3.2 \times 10^{-6}$ M respectively). As with the family lines, *raf*-3T3 was also relatively sensitive to cytosine arabinoside ($ID_{50}$ $2.3 \times 10^{-8}$ M versus $6.0 \times 10^{-7}$ M). The *raf/myc*-3T3 double transfectant did not behave like the *raf*-3T3 with m-AMSA, having a greater sensitivity than control ($ID_{50}$ $1.8 \times 10^{-9}$ M), but showed a similar resistance to VM26 ($ID_{50}$ $3.4 \times 10^{-9}$ M).

**Table I** Cytoplasmic catenation activity (catenanes % total plasmid)

| | *Extract concentration (cell equivalent × $10^5$)* | | | | | |
|---|---|---|---|---|---|---|
| *Line* | *0* | *0.03* | *0.1* | *0.3* | *1.0* | *3.0* |
| Proband | 0 | 0 | 0 | 0 | 5.9±3.5 | 17.1±2.4 |
| (*n*) | (6) | (6) | (6) | (9) | (3) | (3) |
| Great uncle | 0 | 0 | 0 | 0.9±0.6 | 14.4±2.6 | 24.7±4.9 |
| (*n*) | (6) | (6) | (6) | (9) | (3) | (3) |
| Father | 0 | 0 | 0 | 0.6±0.3 | 0 | 11.1±2.6 |
| (*n*) | (6) | (6) | (6) | (9) | (3) | (3) |
| Spouse | 0 | 0 | 0.3±0.2 | 2.2±1.9 | 2.5±1.2 | 1.6±0.5 |
| (*n*) | (6) | (6) | (6) | (9) | (3) | (3) |
| AT patient | 0 | 0 | 0 | 0 | 0 | 3.9±2.7 |
| (*n*) | (6) | (6) | (6) | (6) | (3) | (3) |
| Control-3T3 | 0 | 8.5±2.2 | 17.1±2.3 | 14.9±2.1 | – | – |
| (*n*) | (6) | (6) | (5) | (5) | | |
| *raf*-3T3 | 0 | 0.3±0.2 | 13.7±2.3 | 26.4±2.3 | – | – |
| (*n*) | (6) | (6) | (5) | (5) | | |
| *raf/myc*-3T3 | 0 | 2.0±1.1 | 14.3±4.8 | 28.0±2.8 | – | – |
| (*n*) | (6) | (6) | (5) | (5) | | |
| *ras*-3T3 | 0 | 2.5±1.7 | 10.1±4.0 | 30.9±7.1 | – | – |
| (*n*) | (6) | (5) | (5) | (5) | | |

Results are mean±s.e.m.

**Figure 4** Growth inhibitory concentrations for a panel of cytotoxic agents (for abbreviations see text). Results are $ID_{25}$ values, obtained by regression analysis of pooled experiments from proband (■), great uncle (▼), spouse (O) and AT line (□).

It also differed in its behaviour with cytosine arabinoside, being more sensitive ($ID_{50}$ 4.8 × $10^{-9}$ M).

To substantiate further the perturbed bioactivity of the enzyme and to investigate the basis of the abnormal response to topoisomerase II inhibitors, we used the SDS/KCL precipitation method to evaluate the formation of DNA/protein complexes in response to two topoisomerase II inhibitors, novobiocin and VM26 (Figure 5a). VM26, like VP16 usually increases the amount of precipitated complexes by trapping topoisomerase II at the stage where the enzyme is covalently linked to DNA (Osheroff, 1989). Novobiocin, on the other hand, usually reduces the amount of complexes and this has been suggested to be due to it having a different inhibitory mechanism (stimulatory effects have sometimes been observed, but these are uncommon). In contrast to NIH-3T3 which shows this expected pattern, with more complexes in the presence of VM26 than novobiocin, all three transformants failed to show this pattern (Figure 5b). The *raf* and *raf/myc* lines actually showed a reversal of the usual pattern with higher complexes with novobiocin than with VM26 (227 ± 92 versus 48.5 ± 8.2% diluent control respectively for the *raf*-line, means ± s.e.m. for three independent experiments, and 122–127% versus 62.8% for the *raf/myc*-line). The ras transformant showed a small but significant increment (137 ± 5% diluent control, mean ± s.e.m. of three experiments) in DNA/protein complex formation when treated with VM26 but no

**Figure 5** Influence of novobiocin and VM26 on SDS/KCl precipitation of DNA/protein complexes. (**a**) The amount of protein-linked DNA recovered expressed as per cent diluent control (novobiocin = white columns; VM26 = black columns). (**b**) Per cent difference VM26-novobiocin±s.e.m.

significant change when treated with novobiocin (156 ± 101% diluent control, mean ± s.e.m. three experiments).

Although debated (see below), an influence of DNA topoisomerase on sublethal repair provides a potential link between *ras* and *raf* activity and radioresistance. We therefore examined the relationship between the extent of activation of topoisomerase II and the extent of radioresistence (assessed as $D_q$ values). There was a significant positive correlation (Figure 6) between the percentual increase (with respect to appropriate control) in topoisomerase II activity at high cell extract concentration (mean values from Table I) and the percentual increase in $D_q$ value. $D_{10}$ values (legend to Figure 6) and $D_0$ values (published previously) which reflect

**Figure 6** Correlation between radiation sensitivity (assessed as $D_q$ value) and topoisomerase catenation activity, both expressed with respect to control (spouse for human lines and NIH-3T3 for murine transformants). Absolute values for topoisomerase activity were those given for the highest cell extract concentration tested, in Table I. Equivalent $D_{10}$ values were: spouse 435±3; father 492±9; proband 437±23; great-uncle 564±35; NIH-3T3 427±6; *raf*-3T3 541±35; *ras*-3T3 580±5 these were not significantly correlated to topoisomerase activity. It is essential to normalise with respect to the human and murine controls, since they varied, and it thus allows pooling of human and murine results.

different features of radiation response were not significantly correlated with topoisomerase activity.

### Discussion

Cells from three members of a cancer-prone family with the Li-Fraumeni syndrome had a similar disturbance of dose-response curves for cytoplasmic extract catenation activity. The key features: higher thresholds, steeper slopes and significantly elevated activity levels with respect to controls at higher extract concentrations, were reproduced by transfection of the family's c-*raf-1* oncogene in NIH-3T3 cells. The perturbation of catenation activity is not consistent with there merely being differences in the same enzymatic activity between the cell lines, but indicates modulation of the topoisomerase II activity by an additional factor or factors in test and/or control extracts. Since c-*raf-1* encodes a serine/threonine kinase and this class of enzyme is known to activate the topoisomerases (Durban *et al.*, 1983; Rottmann *et al.*, 1987), the activated c-*raf-1* gene is the obvious candidate for such a factor. Topoisomerase I activity and other DNA nicking agents, detected by relaxation of DNA supercoiling, were not disturbed and the contrasting results with the topoisomerase I inhibitor camptothecin and the topoisomerase II inhibitors in the cytotoxicity test are consistent with these enzyme assay results.

The differential effect of the topoisomerase II inhibitors VP-16, VM-26 and m-AMSA on the family's cells and controls corroborates the evidence for a functional disturbance of topoisomerase II (the difference between the effects of o-AMSA and m-AMSA is consistent with their inhibitory action in the cytotoxicity assay being related to their anti-topoisomerase action). Although resistant to epipodophyllotoxins, the family's cells do not exhibit classical multi-drug resistance since they are not significantly resistant to adriamycin (Ueda *et al.*, 1987). The discrepancy between the results for the two antimetabolities cytosine arabinoside and 6-thioguanine is obscure. Toxicity testing of the murine *raf*-transfectant corroborated these results, including the sensitivity to cytosine arabinonside. The double *raf*/*myc*-3T3 transfectant did not mirror this behaviour, but this could reflect compound effects of the presence of the two oncogenes in murine cells that do not faithfully reflect the situation in the family's cells.

The SDS/KCL precipitation experiments (Figure 5) provide further evidence of functional abnormality of topoisomerase II activity and suggest abnormal interactions with two topoisomerase II inhibitors with different mechanisms of action, VM26 and novobiocin. This potentially provides an explanation for the reduced sensitivity to topoisomerase II targeting epipodophyllotoxins and intercalators. Both the cytotoxicity data and these inhibitors studies extend the observation of abnormal findings from cytoplasmic to nuclear topoisomerase II, since the end-point is the formation of complexes with, or damage of, DNA. Serine threonine kinase-mediated phosphorylation activates topoisomerase II (Durban *et al.*, 1983; Rottmann *et al.*, 1987), which at first sight might be expected to potentiate drugs acting via this enyzme. However, the consequences of phosphorylation vis a vis drug/enzyme/DNA interactions are unknown.

The opposing findings for AT and the family's cells (Table II) extend not only to radiation resistance, to the radiation induced DNA synthesis delay (Paterson *et al.*, 1985; Houldsworth & Lavin, 1980), apparent topoisomerase II activity (at high cytoplasmic extract concentrations), but also to sensitivity to topoisomerase II targeting cytotoxics, to which the family's cells are resistant and AT cells are reportedly hypersensitive (Henner & Blazka, 1986). This reiterates the counter-intuitive relationship between topoisomerase II activity and drug sensitivity, but, as with the family's cells, in AT topoisomerase II is probably not simply quantitatively changed but qualitatively abnormal with normal or even increased protein levels (Singh & Lavin, 1989). Our finding of low topoisomerase II activity, which corroborates observations of Mohamed *et al.* (1987) and Singh *et al.* (1988), contrasts with those of Smith and Makinson (1989) and Davies *et al.* (1989), who found increased activity in the transformed AT5BIVA line, also in one of two untransformed AT fibroblast lines, but low enzyme content in two lymphoblastoid lines. This may reflect methodological differences in the way various assay systems detect qualitatively abnormal enzyme. The linkage between the phenomena illustrated in Table II is certainly intriguing, it extends to cell line mutants (Evans *et al.*, 1989) and leads us to speculate that the primary lesion in the two syndromes interferes with a cellular 'machine' that is involved in regulating/producing these individual cell features.

If one allows that the perturbations of DNA topoisomerase II activity (both activation and inhibition) associated with observed enhancement in mutation, illegitimate recombination and the introduction of other errors may be causally related, and that an aberrant *c-raf-1* gene may behave like other serine-threonine kinases which have been observed to phosphorylate and hence aberrantly activate DNA topoisomerase II, this provides a readily testable hypothesis to account for the family's cells having a higher than usual mutation rate. We are currently examining phosphorylation status and error rate directly. That both activation and inhibition of the enzyme can increase error proneness, potentially reconciles the paradox of two syndromes (AT and Li-Fraumeni) with opposing findings for topoisomerase activity, sensitivity to topoisomerase inhibitors and contrasting response to radiation damage (Table II), but each having increased mutability.

**Table II** Reciprocal relationship between cell properties

| | *Ataxia telangiectiasia* | *Li-Fraumeni family* |
|---|---|---|
| Radiosensitivity | increased[a] | reduced[b] |
| Cytoplasmic topoisomerase II activity | decreased[c] | increased[d] |
| Nuclear topoisomerase II activity | varied | n.a. |
| Sensitivity to cytotoxic action of topoisomerase II inhibitors | increased[a] | decreased[b] |

[a]Increased radiosensitivity in association with sensitivity to topoisomerase II inhibitors (cleavable complex stabilising type), has also been observed in Chinese hamster ovary cell mutants (Robson *et al.*, 1987; Elkind *et al.*, 1988). [b]Radioresistance is associated with resistance to topoisomerase II inhibitors in murine lymphoma lines (Evans *et al.*, 1989). [c]In this but not all assay systems. [d]At high cell extract concentrations.

The presence of coexisting defects in *myc* and *raf* in the family's cells could provide a greatly increased probability for a diverse range of tumours of many types, and could reflect the complex nature of the pedigree with cancers in all four ancestral lines. The *raf* and *myc* oncogenes are known to interact synergistically in oncogenesis in experimental systems (Rapp *et al.*, 1988).

The influence of *ras* and *raf* transfection on radiosensitivity and our finding that both induced an identical perturbation of the topoisomerase II dose response curve, suggests that this could be associated with the radioresistant phenotype (co-ordinate findings for *raf* and *ras* may be due to *ras* being upstream of *raf* in the same signal transduction pathway (Rapp *et al.*, 1988)). Support for this suggested association comes from (1) the significant correlation observed between the extent of topoisomerase II activation and radiation resistance ($D_q$ values); (2) the contrasting effects of serine/threonine kinases (*raf* and *mos*), tyrosine kinases (*fes* and *abl*) and *myc* on radioresistance (Pirollo *et al.*, 1989), given that the latter do not activate topoisomerases; (3) the association between disturbed response to topoisomerase II inhibitors and radioresistance/sensitivity observed in cell mutants (see footnote to Table II); and (4) the association between a radiosensitive phenotype and apparently decreased/perturbed topoisomerase II activity found in the same studies in AT.

Given the debate concerning the evidence for a role of topoisomerase II in DNA repair, speculation concerning a causal relationship between the derangement of topoisomerase II activity and radioresistance must be extremely tentative. However, controversy mostly concerns UV-induced excision repair and the use of novobiocin, an agent incorrectly assumed to be a specific inhibitor of the enzyme (Downes & Johnson, 1988). It is premature to rule out involvement of topoisomerase in all repair processes. First, as mentioned above, the association in AT of radiosensitivity and qualitatively abnormal topoisomerase II (hypersensitive to topoisomerase II targeting cytotoxic agents (Henner & Balzka, 1986)) is provocative. The repair defect in AT has not yet been elucidated, but misrepair (Debenham *et al.*, 1987) rather than lack of ligation (Lehmann, 1982) of double stranded DNA breaks has been implicated. Misrepair could be a consequence of deranged topoisomerase II activity (Bae *et al.*, 1988). Secondly, the failure of topoisomerase II inhibitors (other than novobiocin which has pleotropic affects) to inhibit repair in some systems (Downes *et al.*, 1987; Synder *et al.*, 1987), is not universal (Dressler & Robinson-Hill, 1987). In any case, such results do not rule out an indirect involvement of topoisomerase II related to its influence on chromatin structure, since this may not be sufficiently rapidly affected by the inhibitors in a large enough proportion of the genome to be revealed in short term experiments. Topoisomerases I and II act within and around transcriptionally active genes (Wu *et al.*, 1988) and such genes do have an enhanced repair capacity (Bohr, 1988). Topoisomerase may be involved in transitions of chromatin structure rather than in maintaining the 'active' chromatin configuration, since the enzyme is not found in DNAase hypersensitive, transcriptionally competent sites after transcription has subsided (Muller *et al.*, 1987). This notion of involvement in transitions is consistent with the observation that novobiocin has its major effect in the repair of inactive rather than active chromatin (Bohr & Hanawalt, 1986).

The association of *raf* and a radioresistant phenotype is not limited to the Li-Fraumeni kindred but has been observed in head and neck cancer (Kasid *et al.*, 1987) and with *raf* and amplified *myc* in small cell lung carcinoma (Carney *et al.*, 1983; Rapp *et al.*, 1988). Since radioresistance occurs with other serine/threonine kinases and *ras* too, our finding may have widespread implications. If radioresistance is due to the aberrant topoisomerase this work could have direct clinical application, since this abnormality is potentially reversible. While the familial contribution to oncogenesis appears to be limited (Fraumeni, 1982), the study of cancer families and the mechanism of their inherited susceptibility to many different forms of cancer should provide insights into some key mechanisms of oncogenesis.

J.M.C. is currently recipient of a Clinical Research Training Fellowship from The Wellcome Trust. The project was funded in part by The Leukaemia Research Fund UK to G.E.F., and National Cancer Institute grant CA45158 and National Foundation Cancer Research Grant HU001 to E.H.C. Fibroblast cell lines from the cancer prone family have been generously provided by Dr W. Blattner, NCI.

### References

ALLEY, M.C, SCUDIERO, D.A., MONKS, A. & 7 others (1988). Feasibility of drug screening with panels of human tumour cell lines using a microculture tetrazolium assay. *Cancer Res.*, **48**, 589.

BAE, Y.S., KAWASAKI, I., IKEDA, H. & LIU, L.F. (1988). Illegitimate recombination mediated by calf thymus topoisomerase II *in vitro*. *Proc. Natl Acad. Sci. USA*, **85**, 2076.

BECH-HANSEN, N.T., SELL, B.M., LAMPKIN, B.C. & 4 others (1981). Transmission of in vitro radioresistance in a cancer-prone family. *Lancet*, **i**, 1335.

BLATTNER, W.A., MCGUIRE, D.B., MULVIHILL, J.J., LAMPKIN, B.C., HANANIAN, J. & FRAUMENI, J.F. (1979). Genealogy of cancer in a family. *J. Am. Med. Assoc.*, **241**, 259.

BOHR, V.P. & HANAWALT, P.C. (1986). Novobiocin does not inhibit DNA repair in an active gene. *Carcinogenesis*, **7**, 1917.

BOHR, V.A. (1988). DNA repair and transcriptional activity in genes. *J. Cell Sci.*, **90**, 175.

CARNEY, D.N., MITCHELL, J.B. & KINSELLA, T.J. (1983). In vitro radiation and chemotherapy sensitivity of established cell lines of human small cell lung cancer and its large cell morphological variants. *Cancer Res.*, **43**, 2806.

CHANG, E.H., PIROLLO, K.F., ZOU, Z.Q. & 7 others (1987). Oncogenes in radioresistant, noncancerous skin fibroblasts from a cancer prone family. *Science*, **237**, 1036.

DAVIES, S.M., HARRIS, A.L. & HICKSON, I.D. (1989). Overproduction of topoisomerase II in an ataxia telangiectasia fibroblast line: comparison with a topoisomerase II-overproducing hamster cell mutant. *Nucleic Acids Res.*, **17**, 1337.

DEBENHAM, P.G., WEBB, M.B.T., JONES, N.J. & COX, R. (1987). Molecular studies on the nature of the repair defect in ataxia telangiectasia and their implications for radiobiology. *J. Cell Sci.*, **6**, 177.

DILLEHAY, L.E., DENSTMAN, S.C. & WILLIAMS, J.R. (1987). Cell cycle dependence of sister chromatid exchange induction by DNA topoisomerase II inhibitors in Chinese hamster V79 cells. *Cancer Res.*, **47**, 206.

DOWNES, C.S., MULLINGER, A.M. & JOHNSON, R.T. (1987). Action of etoposide (VP-16-123) on human cells: no evidence for topoisomerase II involvement in excision repair of UV-induced damage, nor for mitochondrial hypersensitivity in ataxia telangiectasia. *Carcinogenesis*, **8**, 1613.

DOWNES, C.S. & JOHNSON, R.T. (1988). DNA topoisomerase and DNA repair. *BioEssays*, **8**, 179.

DRESSLER, S.L. & ROBINSON-HILL, R.M. (1987). Direct inhibitor of UV-induced DNA excision repair in human cells by novobiocin, coumermycin and nalidixic acid. *Carcinogenesis*, **8**, 813.

DURBAN, E., MILLS, J.S., ROLL, D. & BUSCH, H. (1983). Phosphorylation of purified Novikoff hepatoma topoisomerase I. *Biochem. Biophys. Res. Commun.*, **111**, 897.

ELKIND, M.M., UTSUMI, H., KOSAKA, T., BUDDENBAUM, W., SHIBUYA, M. & SUCIU, D. (1988). Inhibitors of topoisomerase and their action in repair-competent and repair-deficient Chinese hamster cells. *J. Cell Biochem.*, Suppl. **12A**, 286.

EPSTEIN, R.J. (1988). Topoisomerase in human disease. *Lancet*, **i**, 521.

EVANS, H.H., RICANATI, M., HORNG, M.-F. & JAROSLAV, M. (1989). Relationship between topoisomerase II and radiosensitivity in mouse L5178Y lymphoma strains. *Mutation Res.*, **217**, 53.

FINLAY, G.F., BAGULEY, B.C. & WILSON, W.R. (1984). A semiautomated microculture technique for investigating growth inhibitory effects of cytotoxic compounds on experimentally growing carcinoma cells. *Anal. Biochem.*, **139**, 272.

FRANCIS, G.E. (1987). Leukaemogenesis: a postulated mechanism involving tyrosine protein kinase and DNA topoisomerase. *Med. Hypoth.*, **22**, 223.

FRANCIS, G.E., BERNEY, J.J., NORTH, P.S. & 4 others (1987). Evidence for the involvement of DNA topoisomerase II in neutrophil-granulocyte differentiation. *Leukemia*, **1**, 653.

FRAUMENI, J.F. (1982). Genetic factors. In *Cancer Medicine*, Holland, J.F. & Frei, E. (eds), p. 5. Lea and Febiger: Philadelphia.

GAULDEN, M.E. (1987). Hypothesis: some mutagens directly alter specific chromosomal proteins (DNA topoisomerase II and peripheral proteins) to produce chromosome stickiness, which causes chromosome aberrations. *Mutagenesis*, **2**, 357.

HALL, E.J. (1988). *Radiobiology for the Radiologist*, Lippincott: Philadelphia.

HENNER, W.D. & BLAZKA, M.E. (1986). Hypersensitivity of cultured ataxis telangiectasia cells to etoposide. *J. Natl Cancer Inst.*, **76**, 1007.

HOULDSWORTH, J. & LAVIN, M.F. (1980). Effects of ionizing radiation on DNA synthesis in ataxia-telangiectasia cells. *Nucleic Acids Res.*, **8**, 3709.

JAXEL, C., TAUDOU, G., PORTEMER, C., MIRABEAU, G., PANIJEL, J. & DUGUET, M. (1988). Topoisomerase inhibitors induce irreversible fragmentation of replicated DNA in concanavalin A stimulated splenocytes. *Biochemistry*, **27**, 95.

KANEKO, M. & HORIKOSHI, J. (1987). Topoisomerase inhibitors suppressed lithocholic acid-induced promotion of transformation in BALB/375. *Br. J. Cancer*, **56**, 614.

KASID, U., PFEIFER, A., WEICHSELBAUM, R.R., DRITSCHILO, A. & MARK, G.E. (1987). The *raf* oncogene is associated with a radiation-resistant human laryngeal tumour. *Science*, **237**, 1039.

KRASNOW, M.A. & COZARILLI, N.R. (1982). Catenation of DNA rings by topoisomerases: mechanism of control by spermidine. *J. Biol. Chem.*, **257**, 2687.

LEHMANN, A.R. (1982). The cellular and molecular responses of ataxia-telangiectasia cells to DNA damage. In *Ataxia-telangiectasia*, Bridges, B.A. & Harden, D.G. (eds), p. 83. Oxford University Press.

LI, F.P. & FRAUMENI, J.F. (1969). Soft-tissue sarcomas, breast cancer, and other neoplasms, a familial syndrome. *Ann. Intern. Med.*, **71**, 747.

LITTLE, J.B., NOVE, J., DAHLBERG, W.K., TROILO, P., NICHOLS, W.W. & STRONG, L.C. (1987). Normal cytotoxic response of skin fibroblasts from patients with Li-Fraumeni familial cancer syndrome to DNA-damaging agents. *Cancer Res.*, **47**, 4229.

MOHAMED, R., SINGH, S.P., KUMAR, S. & LAVIN, M.F. (1987). A defect in DNA topoisomerase II activity in ataxia-telangiectasia cells. *Biochem. Biophys. Res. Commun.*, **149**, 233.

MULLAR, M.T. (1987). Eukaryotic topoisomerase I and II activity in chromatin: mapping catalytic sites during cell differentiation. *Leukemia*, **1**, 827.

OSHEROFF, N. (1989). Biochemical basis for the interaction of type I and II topoisomerase with DNA. *Pharm. Ther.*, **41**, 223.

OVERBYE, K.M., BASU, S.K. & MARGOLIN, P. (1982). Loss of DNA topoisomerase I activity alters many cellular functions in salmonella typhimurium. *Cold Spring Harbor Symp. Quant. Biol.*, **47**, 785.

PATERSON, M.C, GENTNER, N.E., MIDDLESTADT, M.V., MIRZAYANS, R. & WEINFELD, M. (1985). Hereditary and familial disorders linking cancer proneness with abnormal carcinogen response and faulty DNA metabolism. In *Epidemiology and Quantitation of Environmental Risk in Humans from Radiation and Other Agents*, Castellani, A. (ed.), p. 235. Plenum: New York.

PIROLLO, K.F., GARNER, R., YUAN, S.Y., LI, L., BLATTNER, W.A. & CHANG, E.H. (1989). Raf involvement in the simultaneous genetic transfer of the radioresistant and transforming phenotypes. *Int. J. Radiat. Biol.*, **55**, 783.

POMMIER, Y., ZWELLING, L.A., KAO-SHAN, C.S., WHANG-PENG;, J. & BRADLEY, M.O. (1985). Correlations between intercalator-induced DNA strand breaks and sister chromatid exchanges, mutations, and cytotoxicity in Chinese hamster cells. *Cancer Res.*, **45**, 3143.

POMMIER, Y., KERRIGAN, D. & KOHN, K. (1989). Topological complexes between DNA and topoisomerase II and effects of polyamines. *Biochemistry*, **28**, 995.

RAPP, U.R., CLEVELAND, J.L. & BONNER, T.I. (1988). *Oncogene Handbook*. Elsevier North Holland: Amsterdam.

RENAULT, G., MALVY, C., VENEGAS, W. & LARSEN, A.K. (1987). In vivo exposure to four ellipticine derivatives with topoisomerase inhibitory activity results in chromosome clumping and sister chromatid exchange in murine bone marrow cells. *Toxicol. Appl. Pharmacol.*, **89**, 281.

ROBSON, C.N., HOBAN, P.R., HARRIS, A.L. & HICKSON, I.D. (1987). Cross-sensitivity to topoisomerase II inhibitors in cytotoxic drug-hypersensitive Chinese hamster ovary cell lines. *Cancer Res.*, **47**, 1560.

ROTTMANN, M., SCHRODER, H.C., GRAMZOW, M. & 5 others (1987). Specific phosphorylation of proteins in pore complex-laminae from the sponge Geodia cydonium by the homologous aggregation factor and phorbol ester: Role of protein kinase C in the phosphorylation of DNA topoisomerase II. *EMBO. J.*, **6**, 3939.

TRASK, D.K., DIDONATO, J.A. & MULLER, M.T. (1984). Rapid detection and isolation of covalent DNA/protein complexes: application to topoisomerase I and II. *EMBO. J.*, **3**, 671.

SINGH, S.P., MOHAMED, R., SALMOND, C. & LAVIN, M.F. (1988). A defect in DNA topoisomerase II activity in ataxia-telangiectasia cells. *Nucleic. Acids Res.*, **16**, 3919.

SINGH, S.P. & LAVIN, M.F. (1989). Study of DNA topoisomerase II activity in ataxia-telangiectasia cells. *Carcinogenesis*, **10**, 1215.

SKLAR, M.D. (1988). The ras oncogenes increase the intrinsic resistance of NIH-3T3 cells to ionising radiation. *Science*, **239**, 645.

SMITH, P.J. & MAKINSON, T.A. (1989). Cellular consequences of overproduction of DNA topoisomerase II in an ataxia-telangiectasia cell line. *Cancer Res.*, **49**, 1118.

SNYDER, R.D. (1987). Is DNA topoisomerase involved in the UV excision repair process? New evidence from studies with DNA intercalating and non-intercalating anti-tumor agents. *Photochem. Photobiol.*, **45**, 105.

STERNGLANZ, R., DINARDO, S., VOELKEL, K.A. & 5 others (1981). Mutations in the gene coding for *Eschericia coli* DNA topoisomerase I affect transcription and transposition. *Proc. Natl Acad. Sci. USA*, **78**, 2747.

TSE-DINH, Y.C., WONG, T.W. & GOLDBERG, A.R. (1984). Virus- and cell-encoded tyrosine protein kinases inactivate DNA topoisomerases in vitro. *Nature*, **312**, 785.

UEDA, K., CARDERELLI, C., GOTTESMAN, M.M. & PASTAN, I. (1987). Expression of a full-length cDNA for the human 'MDRI' gene confers resistance to colchicine, doxorubicin, and vinblastine. *Proc. Natl Acad. Sci. USA*, **84**, 3004.

WU, H.V., SHYY, S., WANG, J.C. & LIU, L.F. (1988). Transcription generates positively and negatively supercoiled domains in the template. *Cell*, **53**, 433.

YOUNG, R.C., OZOLS, R.F. & MYERS, C.E. (1981). The antracycline antineoplastic drugs. *N. Engl. J. Med.*, **305**, 139.

ZWELLING, L.A., CHAN, D., HINDS, M., SILBERMAN, L. & MAYES, J. (1988). Anion dependent modulations of DNA topoisomerase II mediated reactions in potassium containing solutions. *Biochem. Biophys. Res. Commun.*, **152**, 808.